# Suppressive Effects of Vascular Endothelial Growth Factor-B on Tumor Growth in a Mouse Model of Pancreatic Neuroendocrine Tumorigenesis

**DOI:** 10.1371/journal.pone.0014109

**Published:** 2010-11-24

**Authors:** Imke Albrecht, Lucie Kopfstein, Karin Strittmatter, Tibor Schomber, Annelie Falkevall, Carolina E. Hagberg, Pascal Lorentz, Michael Jeltsch, Kari Alitalo, Ulf Eriksson, Gerhard Christofori, Kristian Pietras

**Affiliations:** 1 Department of Biomedicine, Institute of Biochemistry and Genetics, University of Basel, Basel, Switzerland; 2 German Cancer Research Center Heidelberg (DKFZ), Heidelberg, Germany; 3 Molecular and Cancer Biology Program, Biomedicum, University of Helsinki, Helsinki, Finland; 4 Division of Vascular Biology, Department of Medical Biochemistry and Biophysics, Karolinska Institutet, Stockholm, Sweden; 5 Ludwig Institute for Cancer Research Ltd, Stockholm Branch, Karolinska Institutet, Stockholm, Sweden; University of Texas M. D. Anderson Cancer Center, United States of America

## Abstract

**Background:**

The family of vascular endothelial growth factors (VEGF) contains key regulators of blood and lymph vessel development, including VEGF-A, -B, -C, -D, and placental growth factor. The role of VEGF-B during physiological or pathological angiogenesis has not yet been conclusively delineated. Herein, we investigate the function of VEGF-B by the generation of mouse models of cancer with transgenic expression of VEGF-B or homozygous deletion of *Vegfb*.

**Methodology/Principal Findings:**

Ectopic expression of VEGF-B in the insulin-producing β-cells of the pancreas did not alter the abundance or architecture of the islets of Langerhans. The vasculature from transgenic mice exhibited a dilated morphology, but was of similar density as that of wildtype mice. Unexpectedly, we found that transgenic expression of VEGF-B in the RIP1-Tag2 mouse model of pancreatic neuroendocrine tumorigenesis retarded tumor growth. Conversely, RIP1-Tag2 mice deficient for *Vegfb* presented with larger tumors. No differences in vascular density, perfusion or immune cell infiltration upon altered *Vegfb* gene dosage were noted. However, VEGF-B acted to increase blood vessel diameter both in normal pancreatic islets and in RIP1-Tag2 tumors.

**Conclusions/Significance:**

Taken together, our results illustrate the differences in biological function between members of the VEGF family, and highlight the necessity of in-depth functional studies of VEGF-B to fully understand the effects of VEGFR-1 inhibitors currently used in the clinic.

## Introduction

The formation of new blood vessels, angiogenesis, is a complex and tightly regulated process governed by the action of endogenous pro- and anti-angiogenic factors [1]. The members of the vascular endothelial growth factor (VEGF) family represent prototypical inducers of blood and lymph vessel formation. However, despite our growing knowledge of the molecular cues involved in shaping a new vasculature, the regulation of physiological and pathological blood vessel formation by VEGFs is still not completely understood. The VEGF family is comprised of five members that bind and activate three receptor tyrosine kinases (VEGFR-1, -2 and -3) with different specificity [2]. Haploinsufficiency of *Vegfa* in mice provides an illustrative example of the importance of VEGF-A signaling through VEGFR-1 and -2 for proper endothelial cell function [3], [4]. Placental growth factor (PlGF) binds exclusively to VEGFR-1, and targeting of PlGF inhibits angiogenesis in various pathological settings, including tumor growth [5]. Furthermore, through binding to VEGFR-3 on lymphatic endothelial cells, VEGF-C and -D predominantly regulate lymphangiogenesis [6], even though VEGFR-3 expression by tumor blood vessels has also been reported [7]. VEGF-B specifically binds and activates VEGFR-1, either alone or in conjunction with the co-recpetor neuropilin-1. However, the function of VEGF-B signaling in the context of pathological angiogenesis remains elusive [8].

VEGF-B was first identified as an endothelial cell mitogen highly expressed in heart and skeletal muscle [9]. Consequently, transgenic expression of VEGF-B through adenoviral delivery readily induces angiogenesis in the myocardium [10]. However, VEGF-B deficient mice do not display any overt vascular abnormalities in the unchallenged heart vasculature, even though an impaired recovery from cardiac ischemia is suggestive of an underlying vascular dysfunction [11], [12]. Moreover, ectopic expression of VEGF-B in skeletal muscle does not induce angiogenesis [10]. Most recently, a role for VEGF-B in the trans-endothelial transport of lipids through regulation of fatty acid transport proteins (FATPs) was described [13]. High expression of VEGF-B is observed in a wide variety of tumors, including colon, breast and kidney carcinoma [14], [15], [16], [17]. Expression of VEGF-B is predictive of lymph node metastasis in breast and colon carcinoma, as well as a prognostic factor for shorter survival in node positive breast cancer patients [14], [17], [18]. Intriguingly, the intratumoral level of VEGF-B correlates with microvessel density in oral squamous cell carcinomas, but is not indicative of angiogenesis in breast carcinoma [14], [19].

In order to shed light on the role of VEGF-B in tumor biology in general, and angiogenesis in particular, we analyzed mice with transgenic expression of VEGF-B, and mice deficient for *Vegfb*, in the context of the multistep tumor progression pathway of pancreatic islet carcinoma in RIP1-Tag2 mice [20]. Unexpectedly, ectopic expression of VEGF-B under the insulin promoter reduced the growth of tumors, whereas mice lacking VEGF-B presented with larger tumors. No gross quantitative differences in the vasculature were observed, neither in tumors nor in normal tissues upon altered VEGF-B gene dosage. However, blood vessel morphology was altered in the sense that transgenic expression of VEGF-B yielded thicker vessels, whereas blood vessels in *Vegfb*-deficient tumors appeared slimmer. Together, the data confirm and extend the notion that the various VEGF family members exert different functions in tissue homeostasis and carcinogenesis. Further in-depth investigations are warranted to delineate the detailed functional contribution of VEGF-B to tumor angiogenesis and tumor progression in order to fully understand the complex clinical effects of agents incorporating inhibitory action against VEGFR-1.

## Results

### Transgenic expression of VEGF-B in pancreatic β-cells alters microvessel morphology

**Figure 1:**
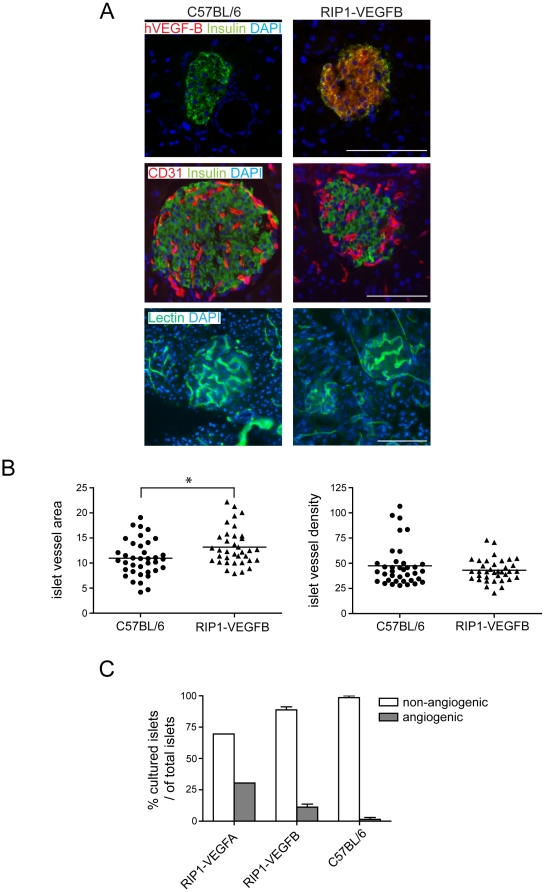
*Characterization of angiogenesis in pancreatic islets from RIP1-VEGFB mice*. A) Pancreatic sections of control C57BL/6 (left) and of RIP1-VEGFB mice (right) were stained for human VEGF-B (red) to detect transgene expression (upper panel), for CD31 (red) to examine intra-insular blood vessel distribution (middle panel) and were perfusion stained with FITC-coupled tomato lectin to evaluate intra-insular blood vessel functionality (lower panel). To visualize islets of Langerhans, pancreatic sections were co-stained with insulin. Nuclei were visualized by DAPI stain. Scale bar: 100 µm. B) Quantification of islet microvessel area and density of C57BL/6 (N =  5, n =  37) and RIP1-VEGFB (N =  4, n =  36) mice. Analysis was performed by determination of the CD31 stained area (left panel) or CD31 counts (right panel) in relation to the islet area using computer-assisted image analysis. * P =  0.0112. N =  number of analyzed mice, n =  number of islets. C) Islets isolated from RIP1-VEGF-A (n = 23, N = 2), RIP1-VEGFB167 (n = 60, N = 10) and C57BL/6 (n = 38, N = 9), mice were co-cultured with HUVEC in a collagen gel matrix and their ability to induce an angiogenic response was determined. The data points represent the average from two independent experiments using C57Bl/6 and RIP1-VEGFB167 mice, while all islets from RIP1-VEGFA mice were analyzed in a single experiment. n =  number of islets, N =  number of mice.

To investigate the role of VEGF-B in normal and pathological angiogenesis, we generated transgenic mice expressing the human VEGF-B_167_ isoform under the control of the rat insulin promoter (RIP1-VEGFB mice), thus directing expression of VEGF-B to the β-cells of the pancreatic islets of Langerhans. Human VEGF-B_167_ activates VEGFR-1 downstream target genes FATP3 and FATP4 to the same extent as mouse VEGF-B_167_ and VEGF-B_186_ isoforms in the mouse pancreatic islet endothelial cell line MS1, indicating that human VEGF-B readily binds mouse VEGFR-1 (Figure S1). Expression of the transgene *in vivo* was confirmed by immunostaining of tissue sections from the pancreas of RIP1-VEGFB mice for human VEGF-B (Figure 1a). No changes were found in the pancreatic islets of transgenic mice in terms of islet architecture, number, or size (Figure S2a-c). Moreover, β-cell density and functionality, as measured by glucose tolerance tests, were normal in RIP1-VEGFB mice (Figure S2d-e). Next, we analyzed the effects of the transgenic expression of VEGF-B on the vascular tree by immunostaining for the endothelial cell marker CD31 and by perfusion with fluorescein-labeled tomato lectin. Whereas there was no difference in the number of islet blood vessels (vascular density; Figure 1a-b), pancreatic islets of RIP1-VEGFB mice exhibited a 20% increase in the fraction of the islet area covered by vessels, as compared to wildtype mice (Figure 1a–b; 13.2±0.6% *vs* 11.0±0.6%, p<0.05). The increase in vessel area was consequent to an apparent increase in the diameter of pancreatic islet microvessels from 8.0±0.25 µm in non-transgenic mice to 9.7±0.50 µm in RIP1-VEGFB mice (Table 1; p<0.01), while vessel length was unchanged (Table 1). No overt differences in perfusion of the islet capillaries were noted (Figure 1a). Finally, to investigate whether islets of Langerhans from RIP1-VEGFB mice exhibited an increased angiogenic potential, we made use of an *ex vivo* collagen gel sprouting assay. Pancreatic islets were purified by limited collagenase digestion of the pancreas, and subsequently seeded into collagen gels together with human umbilical vein endothelial cells (HUVEC). Factors produced by the islet will diffuse into the gel and affect the phenotype of the co-cultured endothelial cells. Islets from RIP1-VEGFA mice were used to demonstrate migration and sprouting of HUVEC towards the islet upon the release of an angiogenic factor (Figure 1c). Whereas 30% of islets from RIP1-VEGFA mice exhibited angiogenic properties, only 13.6% of islets from RIP1-VEGFB mice were able to attract the co-cultured endothelial cells (Figure 1c). No islets from wildtype mice were overtly angiogenic in this assay (Figure 1c).

**Table 1:** Vessel parameters for pancreatic islets or tumors from RIP1-VEGFB, RIP1-Tag2; RIP1-VEGFB, and RIP1-VEGFB^−/−^ mice.

Mouse line	Tissue	Mean vessel length (μm)	Mean vessel diameter (μm)
C57Bl/6	Islets	40.2±2.0	8.0±0.25
RIP1-VEGFB	Islets	43.7±3.8	9.7±0.50*
RIP1-Tag2	Tumors	48.8±2.06	9.3±0.46
RIP1-Tag2; RIP-VEGFB	Tumors	48.9±0.25	11.0±0.64**
RIP1-Tag2; VEGFB^+/−^	Tumors	46.3±1.3	9.8±2.6
RIP1-Tag2; VEGFB^−/−^	Tumors	41.8±1.4§	7.3±0.34***

*p<0.01 *vs* wt; ** p<0.05 *vs* RIP1-Tag2; *** p<0.0001 *vs* RIP1-Tag2; VEGFB^+/−^;

§ p<0.05 *vs* RIP1-Tag2; VEGFB^+/−^.

Taken together, transgenic expression of VEGF-B_167_ in islets of Langerhans increases microvessel diameter, but does not affect islet functionality.

### Transgenic expression of VEGF-B reduces tumor growth in RIP1-Tag2 mice

**Figure 2:**
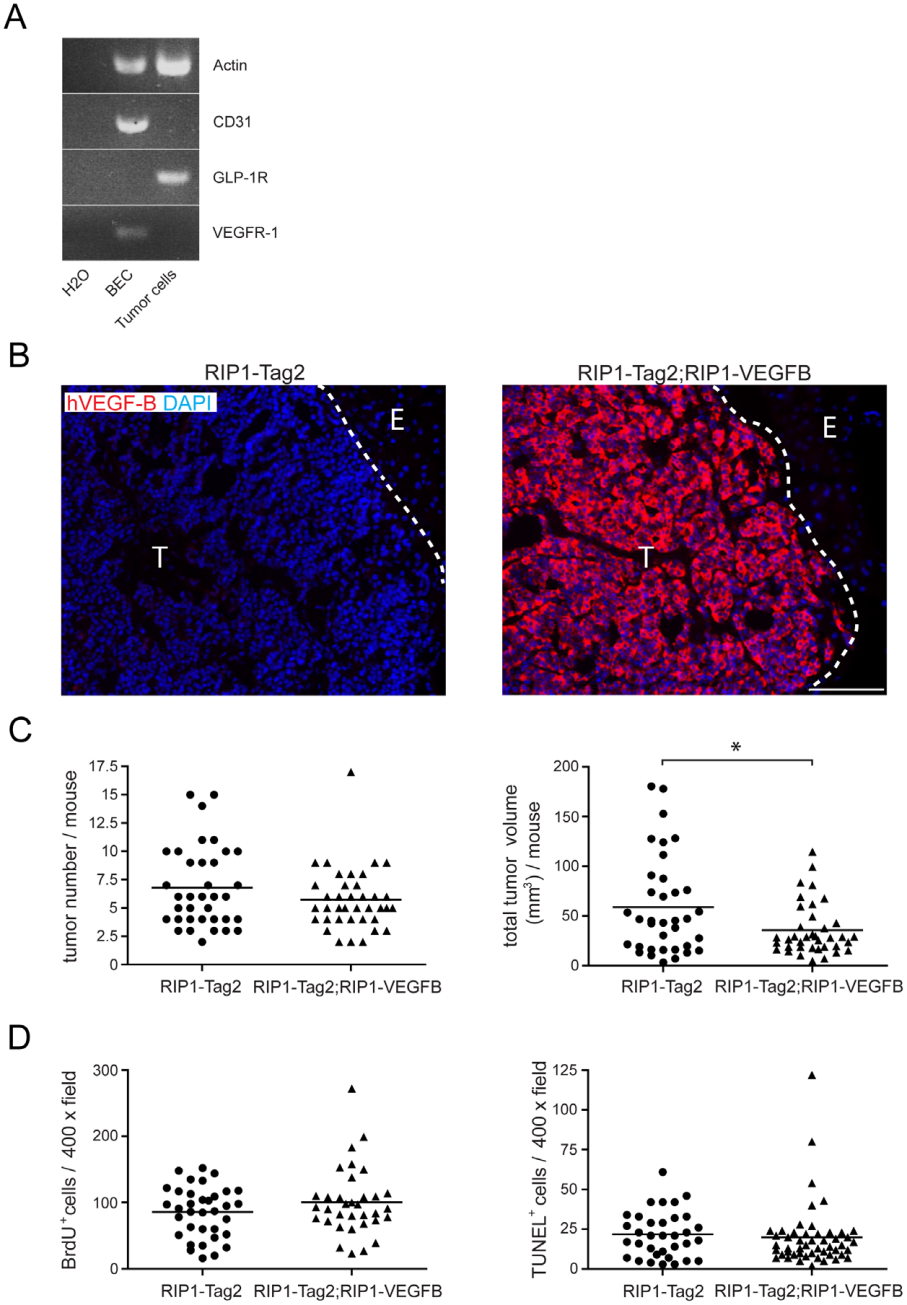
*Characterization of the phenotype of tumors from RIP1-Tag2; RIP1-VEGFB mice*. A) RT-PCR analysis of VEGF-R1 expression in GLP1R+ β-tumor-cells and CD31+ tumor-derived blood-endothelial cells (BEC) isolated from 12 weeks old RIP1-Tag2 mice. B) Pancreatic tumor sections of control RIP1-Tag2 (left) and RIP1-Tag2; RIP1-VEGFB (right) mice were stained for human VEGF-B (red) to detect transgene expression. Nuclei were counterstained with DAPI. T =  Tumor, E =  Exocrine pancreas. Scale bar: 100 µm. C) Tumor incidence (left) and volumes (right) of RIP1-Tag2 (N =  36) and RIP1-Tag2; RIP1-VEGFB (N =  38) mice were determined at the age of 12 weeks. Single points represent the total tumor volume (or tumor number) per mouse as indicated. * P  =  0.0149 (Student's t-test). D) Tumor cell proliferation (left) and apoptosis (right) in RIP1-Tag2 and RIP1-Tag2; RIP1-VEGFB mice was determined by counting the number of BrdU and TUNEL positive tumor cells in a total of 7 to 10 microscopic fields (magnification 400×) per mouse.

The consequences of VEGF-B expression on tumor angiogenesis was assessed in the RIP1-Tag2 mouse model of islet cell carcinoma; a model that has been widely used to study tumor angiogenesis [21], [22], [23], [24], [25], [26], [27]. Quantitative reverse-transcription polymerase chain reaction (qRT-PCR) revealed that VEGF-B is readily detectable in normal β-islets of the mouse pancreas, and maintained at similar levels during the progression through hyperplastic islets and angiogenic islets into overt carcinomas in RIP1-Tag2 mice (data not shown). A cell line established from a RIP1-Tag2 tumor, β-TC3 [28], did not express the *bona fide* receptor for VEGF-B, VEGFR-1, and was not affected in its growth rate by VEGF-B (data not shown). Moreover, tumorous β-cells isolated from RIP1-Tag2 tumors did not express VEGFR-1 mRNA, in contrast to isolated blood endothelial cells from the same tumors (Figure 2a), making it likely that potential effects of transgenic expression of VEGF-B on tumor progression in RIP1-Tag2 mice are caused by paracrine stimulation. Double-transgenic RIP1-Tag2; RIP1-VEGFB mice expressed VEGF-B protein in pancreatic islets at high levels throughout the tumor progression pathway, as determined by immunostaining for human VEGF-B (Figure 2b). Moreover, tumors from RIP1-Tag2; RIP1-VEGFB mice contained abundant levels of human VEGF-B mRNA, as assessed by qRT-PCR, and protein, as assessed by ELISA (Figure S3a–b). No compensatory change was noted in the expression of mouse VEGF-B upon transgenic expression of human VEGF-B (Figure S3a).

While RIP1-Tag2; RIP1-VEGFB mice presented with a similar number of tumors as RIP1-Tag2 mice (Figure 2c, left), expression of the VEGF-B transgene unexpectedly resulted in a significant reduction in total tumor burden by 39% (Figure 2c, right; 59.0±8.2 mm^3^ *vs* 35.7±4.2 mm^3^; p<0.05). No difference in local tumor invasiveness was observed as a consequence of VEGF-B expression (Figure S4a). Next, we analyzed the growth of β-cells in tumor lesions. Neither the proliferative index, as assessed by BrdU incorporation (Figure 2d, left) and phospho-Histone-3 staining (Figure S4b), nor the apoptotic index, as assessed by TUNEL assay (Figure 2d, right) and immunostaining for activated caspase-3 (Figure S4c), was significantly changed in double-transgenic RIP1-Tag2; RIP1-VEGFB mice as compared to single-transgenic RIP1-Tag2 mice. Also, no difference in terms of tumor cell density was observed (Figure S4d). Possibly, transgenic expression of VEGF-B produces subtle changes in the proportion of cells in different cell cycle stages, including quiescent cells in G_0_, thus retarding overall tumor growth. Recently, new roles for VEGF-B in the regulation of pro-apoptotic members of the Bcl-2 family and in the regulation of expression of FATPs in the endothelium were described [10], [29], [30]. However, we found no VEGF-B-dependent changes in the expression of BH3-only proteins, or of FATPs, in whole tumor lysates, and there was no discernible difference in fatty acid accumulation in RIP1-Tag2 lesions upon transgenic expression of VEGF-B (Figure S5a–b).

Thus, expression of VEGF-B in the context of RIP1-Tag2 tumorigenesis significantly retarded tumor growth without affecting the rates of proliferation or apoptosis of β-tumor cells.

### Microvessels of RIP1-Tag2 tumors have a thicker diameter upon transgenic expression of VEGF-B

**Figure 3:**
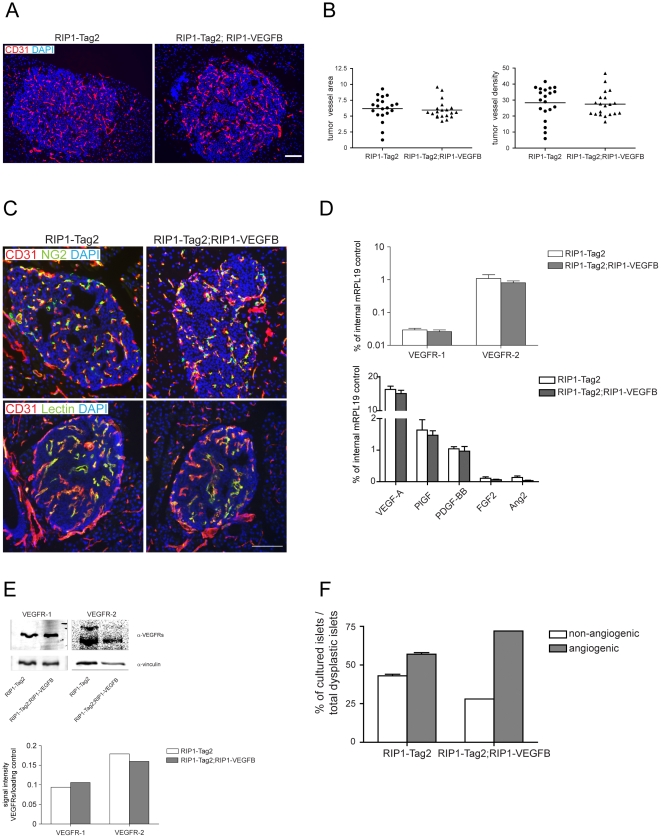
*Characterization of the vascular and angiogenic profile of tumors derived from RIP1-Tag2; RIP1-VEGFB mice*. A) Representative immunofluorescence microphotographs of pancreatic tumor sections of RIP1-Tag2 (left) and RIP1-Tag2; RIP1-VEGFB (right) mice stained for CD31. Scale bar: 100 µm. B) Quantification of intratumoral vessel density in RIP1-Tag2 (N =  5, n =  20) and RIP1-Tag2; RIP1-VEGFB (N =  5, n =  20) was performed using computer-assisted image analysis. Results are displayed as relation of CD31 stained area or CD31 positive cell counts to tumor area. N =  number of analyzed mice, n =  number of islets. C) Representative immunofluorescence microphotographs of pancreatic tumor sections of RIP1-Tag2 (left) and of RIP1-Tag2; RIP1-VEGFB (right) mice double-stained with CD31 and the pericyte marker NG2 (upper panel) or with FITC-coupled tomato lectin (lower panel). Scale bar: 100 µm. D) Evaluation of mVEGFR-1, mVEGFR-2, mVEGF-A, mPlGF, mPDGF-BB, mFGF2 and mAng2 mRNA expression by quantitative RT-PCR in total tumors of RIP1-Tag2 (n = 5) and RIP1-Tag2; RIP1-VEGFB (n = 5) mice. The mRNA expression profiles of the indicated genes are normalized to the expression of the internal control gene ribosomal protein 19 (mRPL19). E) Upper panel: Immunoblot for mVEGFR-1, mVEGFR-2 and vinculin (loading control) of total tumor lysate prepared from pancreatic tumors of a 12 weeks-old RIP1-Tag2 and RIP1-Tag2; RIP1-VEGFB. Lower panel: Quantitation. F) Determination of the percentage of dysplastic islets isolated from RIP1-Tag2 (white bar, n =  24) and RIP1-Tag2; RIP1-VEGFB mice (grey bar, n =  25) which are able to induce an angiogenic response when they were co-cultured with HUVECs in a collagen gel matrix.

To assess whether VEGF-B, by signaling through its receptor VEGFR-1 on endothelial cells, affects the angiogenic phenotype of RIP1-Tag2 tumors, we analyzed vascular parameters in RIP1-Tag2; RIP1-VEGFB mice. Immunostaining for the endothelial cell marker CD31 revealed no difference in the blood vessel content of VEGF-B-expressing lesions, compared to control lesions (Figure 3a–b). In keeping with the increase in vessel diameter of pancreatic islets in RIP1-VEGFB mice, tumors from RIP1-Tag2; RIP1-VEGFB mice exhibited a similar thickening of microvessels compared to tumors from RIP1-Tag2 mice (Table 1; 11.0±0.64 µm *vs* 9.3±0.46 µm, p<0.05). Furthermore, the extent of NG2^+^ pericyte coverage of tumor microvessels (RIP1-Tag2: 94.3% ± 0.87% vs RIP1-Tag2; RIP1-VEGFB: 94.3% ± 0.62% of all vessels were covered with NG2), as well as the functionality of the vasculature, as quantified by perfusion with fluorescein-labeled tomato lectin (RIP1-Tag2: 93.1% ± 1.4% vs RIP1-Tag2; RIP1-VEGFB: 93.5% ± 1.4% of all vessels were lectin perfused), remained unaffected by the expression of VEGF-B (Figure 3c).

To assess whether the gross angiogenic profile was changed upon transgenic expression of VEGF-B, we analyzed the expression of VEGFRs and of prototypical angiogenic factors in RIP1-Tag2 tumors by qRT-PCR. Neither the expression of other VEGF family members, such as VEGF-A and PlGF, nor the expression of PDGF-BB, FGF2 or Angiopoietin-2, was altered by the presence of the VEGF-B transgene (Figure 3d). Moreover, the protein levels of VEGFR-1 and VEGFR-2 remained unchanged upon ectopic VEGF-B expression in RIP1-Tag2 tumors (Figure 3e). Finally, islets from 12-weeks old RIP1-Tag2 mice were embedded in collagen to ascertain whether they harbored angiogenic properties *ex vivo*. Islets from single-transgenic RIP1-Tag2 mice were deemed to be overtly angiogenic in 57% of the cases (Figure 3f). Expression of VEGF-B in the purified islets marginally increased the incidence of angiogenic islets to 72% (Figure 3f).

In summary, while producing an increased thickness of tumor microvessels, expression of VEGF-B neither affected vessel abundance, architecture and function, nor angiogenic factor profile in tumors of RIP1-Tag2 mice.

### Immune cell infiltration is not altered by expression of VEGF-B in RIP1-Tag2 lesions

**Figure 4:**
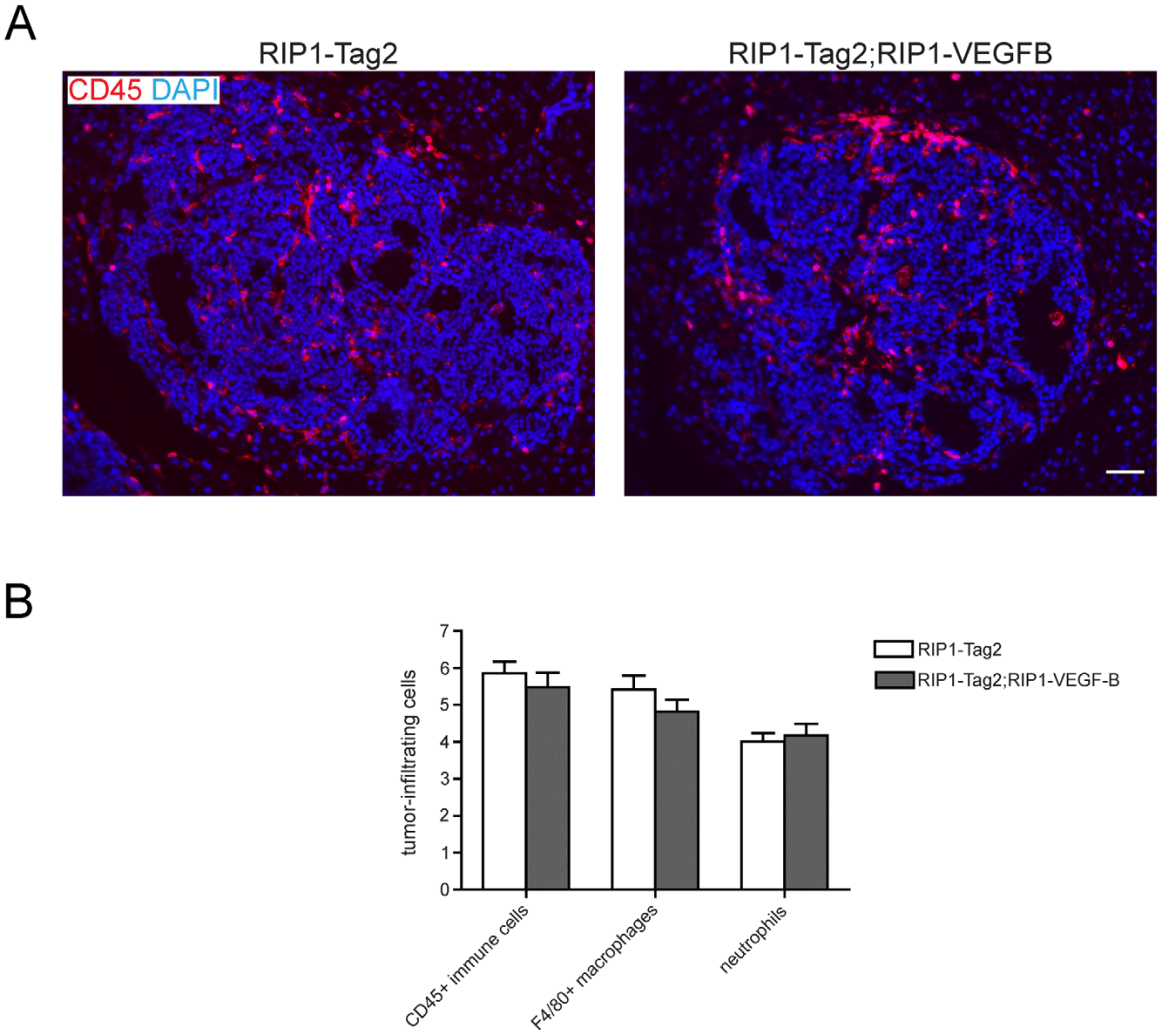
*Analysis of inflammatory cell infiltration in tumors derived from RIP1-Tag2; RIP1-VEGFB mice*. A) Representative immunofluorescence microphotographs of pancreatic tumor sections of RIP1-Tag2 (left) and RIP1-Tag2; RIP1-VEGFB (right) mice stained for CD45. Scale bar: 100 µm. B) Quantification of tumor-infiltrating immune cells in RIP1-Tag2 (white bar, N =  5, n =  26-46) and RIP1-Tag2; RIP1-VEGFB (grey bar, N =  5, n = 27–45). The number of tumor-infiltrating CD45+ and F4/80+ cells as well as 7/4+ neutrophils was determined by quantification of the area stained for the selected marker in relation to the total tumor area using computer-assisted image analysis. N =  number of analyzed mice, n =  number of tumors.

Apart from its role in endothelial cell biology, VEGFR-1 is also expressed by various cells of the immune system, including macrophages [31] and hematopoietic progenitor cells [32]. Thus, we appraised the effects of transgenic expression of VEGF-B on the immune cell infiltration of RIP1-Tag2 lesions. As determined by immunostaining for CD45, there was no gross difference in the abundance of leukocytes in RIP1-Tag2; RIP1-VEGFB mice compared to wildtype RIP1-Tag2 mice (Figure 4a-b). Specifically, both macrophages and neutrophils have been implicated in the growth and angiogenesis of RIP1-Tag2 tumors [33], [34]. However, the infiltration of macrophages and neutrophils, as evaluated by immunostaining for F4/80 and Gr1, respectively, was not altered by the expression of VEGF-B in RIP1-Tag2 double transgenic mice (Figure 4a-b).

### RIP1-Tag2 mice genetically deficient for VEGF-B present with larger tumors

**Figure 5:**
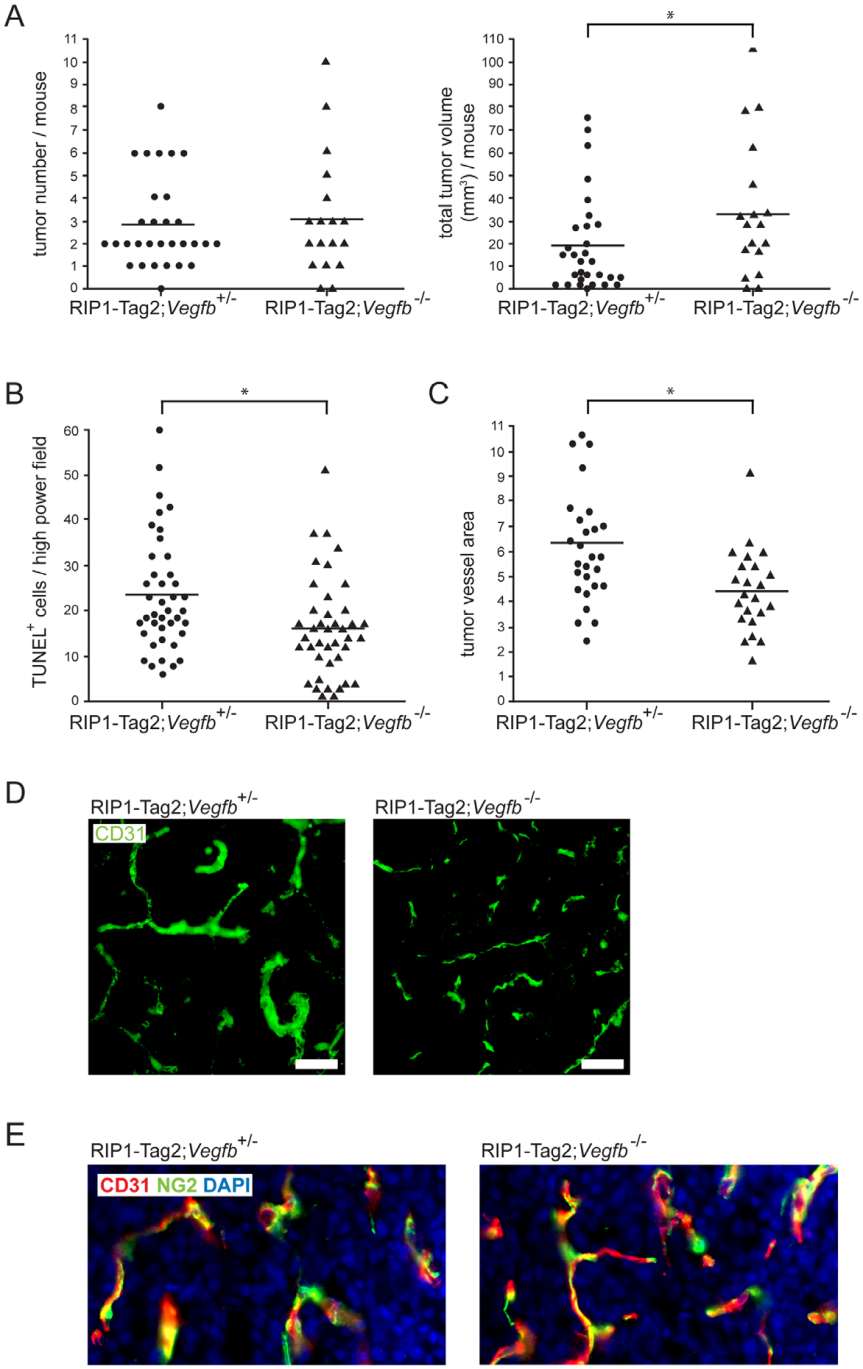
*Characterization of the tumor and angiogenic phenotype of tumors derived from Vegfb-deficient RIP1-Tag2 mice*. A) Quantification of tumor number (left) and total tumor burden (right) of 12 weeks-old RIP1-Tag2; *Vegfb* ^+/−^ (n = 30) and RIP1-Tag2; *Vegfb* ^−/−^ (n = 18) mice. B) Quantification of tumor cell apoptosis in lesions (n = 40 for each genotype) from RIP1-Tag2; *Vegfb* ^+/−^ and RIP1-Tag2; *Vegfb* ^−/−^ mice, as assessed by TUNEL assay. C,D) Vascular density (C) and morphology (D) in lesions from RIP1-Tag2; *Vegfb* ^+/−^ (n = 27) and RIP1-Tag2; *Vegfb* ^−/−^ (n = 23) mice. Scale bar, 50 µm. E) Pericyte coverage (n = 24 for each genotype), as visualized by immunostaining for the pericyte marker NG2 (green) in relation to the endothelial cell marker CD31 (red). Cell nuclei were visualized using DAPI (blue).

To validate the unexpected reduction in tumor burden observed following transgenic expression of VEGF-B in pancreatic islets, we generated RIP1-Tag2 mice deficient for VEGF-B expression (RIP1-Tag2; *Vegfb* ^−/−^). Initial analyses of the phenotype of 12-weeks old RIP1-Tag2; *Vegfb* ^−/−^ demonstrated that the loss of VEGF-B affected neither the number of pre-malignant angiogenic islets, nor the number of tumors (Figure S6a and Figure 5a, left). However, in agreement with the reduced tumor burden of RIP1-Tag2; RIP1-VEGFB mice, the average tumor volume of RIP1-Tag2; *Vegfb* ^−/−^ mice was increased by 81% compared to RIP1-Tag2; *Vegfb* ^+/−^ (33.6±29.9 mm^3^ *vs* 18.6±21.3 mm^3^; Figure 5a, right; p<0.05). Tumorous β-cells of the pancreas exhibited a significantly lower degree of apoptosis, as assessed by the TUNEL assay, in RIP1-Tag2; VEGFB^−/−^ mice compared to RIP1-Tag2; *Vegfb* ^+/−^ mice, consistent with the increased tumor size (Figure 5b; p<0.05). There was no difference in the fraction of tumor cells that expressed the proliferative marker Ki67 (Figure S6b). Next, we analyzed the vascular tree of pancreatic lesions from RIP1-Tag2; *Vegfb* ^−/−^ mice. The fraction of tumor area covered by CD31^+^ endothelial cells was significantly lower in mice deficient for *Vegfb* (Figure 5c; p<0.05). Nevertheless, the vessel density (number of vessels per high power field) was similar, regardless of *Vegfb* gene dosage (data not shown) and vessel length was only marginally decreased (Table 1; p<0.05). Strikingly, microvessels of RIP1-Tag2; *Vegfb* ^−/−^ lesions were thinner than in VEGF-B proficient tumors (Figure 5d and Table 1; mean vessel diameter 7.3±0.34 µm *vs* 9.8±2.6 µm; p<0.0001), accounting for the reduced total vessel surface area. However, no change in the extent of pericyte coverage due to the absence of VEGF-B was detected by immunostaining for the pericyte marker NG2 (RIP1-Tag2; *Vegfb* ^+/−^: 94% ± 2.1% vs RIP1-Tag2; *Vegfb* ^−/−^: 92.3% ± 3.1% of all vessels were covered with NG2) (Figure 5e). Finally, neither immune cell infiltration, as assessed by quantification of CD45^+^ cells within RIP1-Tag2 tumors, nor lipid accumulation was different in RIP1-Tag2; *Vegfb* ^−/−^ mice compared to RIP1-Tag2; *Vegfb* ^+/−^ mice (Figure S6c and data not shown).

Thus, in agreement with the reduced tumor size upon transgenic expression of VEGF-B in tumorous pancreatic β-cells, *Vegfb-*deficient RIP1-Tag2 mice present with larger tumors that harbor morphological changes in the vascular bed.

## Discussion

A definitive role for VEGF-B in tumor biology has thus far not been defined, and there is an apparent paucity of pre-clinical studies investigating the function of tumor-derived VEGF-B. Our finding that VEGF-B gene dosage correlates inversely with tumor growth was unexpected in the light of the prominent and well-documented role of other members of the VEGF family in tumor angiogenesis. Specifically in RIP1-Tag2 mice, overexpression of VEGF-A accelerates tumor progression and growth by facilitating activation of the angiogenic switch [35]. On the other hand, depletion of VEGF-A in the pancreatic β-cells by genetic means essentially eliminates tumor progression beyond the angiogenic stage [21]. The diversity of outcomes in functional studies of VEGF family members is further highlighted by the fact that neutralization of PlGF or blockade of VEGFR-1 in RIP1-Tag2 mice does not affect tumor angiogenesis or growth [26], [36]. To understand the mechanism behind the observed outcomes, the VEGFR-1 and VEGFR-2 occupancy by all different VEGF ligands in the context of ligand over-expression or deficiency have to be considered. In the present study, the transgenically expressed VEGF-B in RIP1-Tag2 islets possibly displaced VEGF-A and PlGF from VEGFR-1, thus diminishing signaling by overtly pro-angiogenic factors. Conversely, the absence of VEGF-B in RIP1-Tag2; *Vegfb* ^−/−^ mice may enhance the specific signaling by VEGF-A and PlGF through VEGFR-1 in endothelial cells. Moreover, members of the VEGF family have been reported to form heterodimers, the abundance and activity of which is presently still unknown [37], [38], [39]. Additional complexity comes from possible effects on the competitive binding equilibrium of the VEGF family co-receptors neuropilin-1 and -2 [40], which also act as an integral part of the semaphorin and plexin family of angiogenesis regulators [41]. Clearly, more in-depth studies of VEGFR ligand and receptor/co-receptor occupancy following various pharmacological and/or genetic perturbations of the VEGF system are warranted together with functional studies aimed at revealing ligand-specific signaling effectors downstream of VEGFR-1.

A number of possible explanations for the observed effects of VEGF-B on tumor growth relating to cell metabolism or energy usage are discussed below. Firstly, VEGF-B was recently attributed a role in metabolism by controlling the trans-capillary transport of long-chain fatty acids through transcriptional regulation of FATPs in the endothelium [10], [30]. Thus, the release of VEGF-B would allow tissues with a high metabolic turnover, including tumors, to meet their demand for fuel. In the context of pancreatic β-cell biology, excess lipid exposure is known to be detrimental for both β-cell function and survival [42]. Moreover, free fatty acids induce the production of the vasodilator nitric oxide in pancreatic islets [43]. Interestingly, indications of diminished lipotoxicity in tumors from RIP1-Tag2; *Vegfb* ^−/−^ mice come from the observations of a decreased rate of β-cell apoptosis and a constricted vasculature. However, we did not observe increased β-cell apoptosis or tumoral lipid accumulation and fatty acid transporter protein expression in RIP1-Tag2; RIP1-VEGFB tumors. Thus, it is unlikely that the observed changes in tumor growth rate result solely from changes in lipid accumulation and toxicity. Secondly, tumors preferentially meet their energy demand through glycolysis [44]. Studies of patients with colorectal carcinoma using positron emission tomography (PET) demonstrate an inverse correlation between the expression of VEGF-B and k3, the most important kinetic parameter for determining uptake of the glucose analog ^18^F-FDG [45]. Whether the observed VEGFB-dependent changes in tumor growth rate in the RIP1-Tag2 model are due to an altered supply or utilization of glucose warrants further analysis by a PET-based approach.

Pharmacological inhibitors of VEGF-signaling have now made their way into the clinic following successful pre-clinical studies. However, the patient benefit is comparatively modest and is typically measured in months [46], [47], [48]. The mechanism of action of VEGF inhibitors is still debated and has been suggested to include overt anti-angiogenic actions, vessel “normalization”, inhibition of mobilization of endothelial precursor cells, suppression of intra-tumoral regulatory T-cells, as well as direct effects on tumor cells [49]. Given the diversity and complexity of signaling derived from the VEGF ligand/receptor system revealed by this and other studies, it is important to fully understand the contributions of each component. Despite the observed inhibition of tumor growth in the present study, human tumors, as well as wildtype RIP1-Tag2 tumors, readily express VEGF-B, indicative of functional significance. Again, it may be that a balance between the three different VEGFR-1 ligands must be maintained to achieve optimal conditions for endothelial cell function and growth. Our results raise the possibility that indiscriminate blocking of VEGF signaling may lead to a mix of favorable and detrimental effects in terms of net tumor growth. Thus, further pre-clinical as well as clinical studies on the role of VEGF-B in tumor biology in the context of pharmacological inhibition of VEGF family signaling are justified in order to maximize patient benefit.

## Materials and Methods

### Mice

All experimental procedures involving mice were approved by and performed according to the guidelines and regulations of the local committees for animal care (the Swiss Federal Veterinary Office (SFVO) - the Cantonal Veterinary Office of Basel Stadt, permit numbers 1878, 1907, 1908, and Stockholm North committee for animal experimentation, permit number N146/08) and all efforts were made to minimize suffering.

The RIP1-VEGFB vector encodes full length human VEGF-B167 (accession number EMBL: U43369) under the control of the rat insulin gene II promoter (RIP1; [20]. For generation of transgenic RIP1-VEGFB mice, a RIP1-VEGFB fragment, obtained by BamH1 digestions of the RIP1-VEGFB vector was injected into the pronucleus of fertilized C57BL/6 oocytes according to standard protocol [50]. The genotypes of founder animals were confirmed by PCR analysis of genomic DNA using the primer pair 5′-TAA TGG GAC AAA CAG CAA AG-3′ and 5′-CGC GAG TAT ACA CAT CTA TCC-3′.

Double-transgenic RIP1-Tag2; RIP1-VEGFB mice were obtained by crossing the single-transgenic RIP1-VEGFB mice with RIP1-Tag2 mice. All mice were kept on C57BL/6 background. RIP1-Tag2 mice deficient mice were obtained by crossing RIP1-Tag2 mice with homozygous null mice for *Vegfb*, after which the heterozygote offspring was again backcrossed to homozygous null mice [11], [12].

Phenotypical analysis of all mice and their littermates were performed between the age of 10 and 12 weeks. Tumor incidence per mouse was determined by counting the numbers of macroscopically visible pancreatic tumors with a diameter above 1 mm. Tumor volume was calculated from the measured tumor diameter assuming a spherical tumor shape. To measure tumor cell proliferation using bromodeoxyuridine, mice were injected intraperitoneally with 100 µg bromodeoxyuridine (Sigma) 90 min prior to sacrificing of the animals.

To evaluate vessel functionality, anesthetized mice were tail vein-injected with 100 µl of 1 mg/ml fluorescein-labeled *Lycopersicon esculentum* lectin (Vector Laboratories). After 5 min, mice were heart-perfused consecutively with 10 ml 4% paraformaldehyde and 10 ml PBS, and subsequently the pancreata were isolated.

### Histopathological analysis

The isolated mice pancreata were either directly embedded in OCT compound (Tissue Tek, Redding, CA) and snap frozen in liquid nitrogen or fixed (2 hours in 4% paraformaldehyde followed by incubation in 12%, 15%, 18% sucrose for 1 h each and 30% sucrose O/N) before OCT embedding. For paraffin embedding, the pancreata were fixed overnight in 4% paraformaldehyde and dehydrated prior embedding. Immunostaining was performed on paraffin sections (5 µm) or on cryosections (7 µm) as previously described [25], [35].

The following antibodies were used: rat anti-mouse CD31 and rat anti-mouse CD45 (BD Pharmingen, Franklin Lakes, NJ), goat anti-human VEGF-B167/186 (R&D Systems), rabbit anti-mouse NG-2 (Chemicon, Hampshire, UK), rat anti-mouse neutrophils (clone7/4), rat anti-mouse F4/80 (AbD Serotech), rabbit anti-Ki67 (Novocastra laboratories, Newcastle, United Kingdom) the in Situ Cell Death Detection Kit, POD (terminal deoxynucleotidyl transferase-mediated nick end labeling (TUNEL); Roche) and biotinylated mouse anti-BrdU (Zymed). Secondary antibodies for immunofluorescence were conjugated either with AlexaFluor 488 or 568 (Molecular Probes). Nuclei were counterstained with 6-diamidino-2-phenylindole (DAPI). Stained pancreata sections were viewed on a Nikon Diaphot 300 immunofluorescence microscope (Nikon, Egg, Switzerland) using Openlab 3.1.7. Software (Improvision, Coventry, England) or on a Nikon Eclipse E800 microscope equipped with Nikon Plan Fluor objectives. Tumor microvessel density as well as the amount of tumor-infiltrating immune cells was quantified using Image J software (Rasband, W.S., ImageJ, U. S. National Institutes of Health, Bethesda, Maryland, USA, http://rsb.info.nih.gov/ij/, 1997–2007) and is displayed as % of stained intratumoral area to tumor area or counts per tumor area. Lectin perfused and pericytes covered tumor blood vessels are shown as % of the total intratumoral vessels. To determine tumor cell proliferation and apoptosis, BrdU/TUNEL/or Ki67 positive nuclei were counted in randomly chosen 40× magnification fields of tumor tissue. Per mouse approximately 10 fields were examined.

Microvessel diameter and length were quantified in C57Bl/6 (n = 233 vessel structures), RIP1-VEGFB (n = 254 vessel structures), RIP1-Tag2 (n = 2034 vessel structures), RIP1-Tag2; RIP1-VEGFB (n = 1811 vessel structures), RIP1-Tag2; *Vegfb*+/− (n = 2806 vessel structures), and RIP1-Tag2; *Vegfb*−/− (n = 1775 vessel structures) using the Volocity Quantitation software package Perkin-Elmer, Waltham, MA). Vessel diameter was taken as object area divided by skeletal length.

### RT-PCR

A single cell suspension of pancreatic tumors of 12 -week -old RIP1-Tag2 mice was prepared by Dispase digestion. For subsequent isolation of GLP-1R+ β-tumor cells and tumor-derived CD31+ blood endothelial cells (BEC) by fluorescence-activated cell sorting (FACS), cells were stained with FITC labeled glucagon-like peptide 1 receptor (GLP-1R) peptide ligand exendin-4 (Phoenix Pharmaceuticals, Inc.) and with APC-CD31 (Biolegend). Total RNA was extracted from isolated cells, cDNA prepared and the expression of VEGF-R1 was evaluated by PCR. The following primers were used:

mActin: ACACTGTGCCCATCTACGAGG and CATGCATGCCACAGGATTCC

mCD31: GGAGTCAGAACCCATCAGGA and TACTGGGCTTCGAGAGCATT

mGLP-1R: TCAGAGACGGTGCAGAAATG and CAAGGCGGAGAAAGAAAGTG

mVEGF-R1: CGGCAGACCAATACAATCCT and CCGCTGCCTTATAGATGCTC.

### Quantitative RT-PCR

Total RNA was extracted from isolated pancreatic tumors of RIP1-Tag2 and RIP1-Tag2; RIP1-VEGFB mice using TRIzol reagent. After DNase treatment of the RNA, first-strand cDNA was synthesized with M-MLV reverse transcriptase RNAse-H (Promega). Quantitative PCR for mouse Fatp1, 3, 4, Bik, Bmf, VEGF-A, PlGF, PDGF-BB, FGF2 and Ang2 transcripts was done on ABI Prism 7000 (Applied Biosystems) using the SYBR-green PCR MasterMix (Applied Biosystems) and normalized versus the mouse ribosomal protein 19 (mRPL19) transcript. The following primers were used:

mRPL19: ATCCGCAAGCCTGTGACTGT and TCGGGCCAGGGTGTTTTT

mVEGF-A: ACTGGACCCTGGCTTTACTG and TCTGCTCTCCTTCTGTCGTG

mPlGF: CTGGGTTGGCTGTGCATT and GGCACCACTTCCACTTCTGT

mPDGF-BB: CGAGGGAGGAGGAGCCTA and GTCTTGCACTCGGCGATTA

mFGF2: CGGCTCTACTGCAAGAACG and TGCTTGGAGTTGTAGTTTGACG

mAng2: CACACTGACCTTCCCCAACT and CCCACGTCCATGTCACAGTA

mVEGFR1: ACCTCCGTGCATGTGTATGA and ATGGACAGCCGATAGGAC

mVEGFR2: AAAGCGGGACGAGGAGAG and CAGGTTGCACAGTAATTTCAG.

### Collagen Gel Assay

Islets from C57BL/6, RIP1-VEGFA and RIP1-VEGFB mice or dysplastic islets from RIP1-Tag2 and RIP1-Tag2; RIP1-VEGFB mice were isolated at the age of 6 and 9 weeks respectively as previously described [51]. The isolated islets were cultured together with human umbilical vein endothelial cells (HUVEC) in a three-dimensional collagen matrix for 2-3 days prior the angiogenic response was analyzed. Approximately 30 islets were scored per genotype.

### Protein Analysis

Tumor lysate from pancreatic tumors of 12 week-old RIP1-Tag2 and RIP1-Tag2; RIP1-VEGFB mice were prepared by mechanical disruption of the tissue in JS lysis buffer (50 mM HEPES pH 7.5, 150 mM NaCl, 1.5 mM MgCl_2_, 5 mM EGTA, 1% TritonX-100, 1% glycerol) containing proteinase inhibitor. Lysates were cleared by centrifugation (12000 xg, 20 min). Subsequently, equal protein amounts of the samples were separated on a SDS-Page, transferred to PVDF membrane and stained for mVEGFR-1 (Epitomics), mVEGFR-2 (Cell Signaling) and vinculin (Santa Cruz). For quantitation fluorescent dye labeled secondary antibodies (Li-Cor) were used. The fluorescent signals was measured with the infrared imager odyssey (Li-Cor) and analyzed by ImageJ software Rasband, W.S., ImageJ, U. S. National Institutes of Health, Bethesda, Maryland, USA, http://rsb.info.nih.gov/ij/, 1997–2007). Protein expression is displayed as the ratio between fluorescent signal intensity of the protein of interest and of the loading control vinculin.

### Statistical analysis

All statistical analyses were performed using a Student's un-paired, two-sided t-test with p<0.05 considered significant.

## Supporting Information

Figure S1 Comparison of the ability of mouse and human VEGF-B to activate VEGFR-1 downstream target gene transcription. Quantitative RT-PCR determination of the induction of FATP3 and FATP4 mRNA by mouse pancreatic islet endothelial cells (MS1) following 24h of stimulation by control, human VEGF-B167, or mouse VEGF-B167 and VEGF-B186.
(0.13 MB TIF)

Figure S2 Characterization of the pancreatic islet architecture in RIP1-VEGFB mice. A) Pancreatic sections of control C57BL/6 (left) and of RIP1-VEGFB mice (right) stained for glucagon and insulin to examine islet architecture. Nuclei were counterstained with DAPI. Scale bar: 100 µm. B, C) Quantification of islet number (B, left), area (B, right) and Beta-cell density (C) was performed on H&E stained paraffin sections of C57BL/6 (N = 8) and RIP1-VEGFB (N = 6) mice. Determination of islet area and of Beta-cell number per islet area was done using computer-assisted image analysis. Beta-cell density is shown as nuclei per islet area in mm2. *, p  =  0.0108 (Student's t-test). D) Intra-peritoneal glucose tolerance test: After 16 hours of starvation C57BL/6 (N = 6) and RIP1-VEGFB (N = 6) mice were i.p. injected with 1g glucose/kg body weight, and subsequently blood glucose levels were determined at the indicated time points.
(3.68 MB TIF)

Figure S3 Analysis of the expression of VEGFB in RIP1-VEGFB mice. A) Quantitative RT-PCR determination of expression of mouse and human VEGF-B in tumors from RIP1-Tag2 and Rip1-Tag2; RIP1-VEGFB mice. B) Analysis of the abundance of human VEGF-B protein in serum and tumor tissue from RIP1-Tag2; RIP1-VEGFB mice using ELISA.
(0.23 MB TIF)

Figure S4 Characterization of the phenotype of tumors derived from RIP1-Tag2; RIP1-VEGFB mice. A) Staging of tumors into normal/hyperplastic islets, adenoma or carcinoma in RIP1-Tag2 (N =  6) and RIP1-Tag2; RIP1-VEGFB (N =  6) mice. B, C, D) Quantification of tumor cell proliferation in RIP1-Tag2 (N =  5, n =  28) and RIP1-Tag2; RIP1-VEGFB (N =  5, n =  38) (B) mice, of tumor cell apoptosis in RIP1-Tag2 (N =  4, n =  23) and RIP1-Tag2; RIP1-VEGFB (N =  5, n =  29) mice (C) and of tumor cell density in RIP1-Tag2 (N =  8, n =  32) and RIP1-Tag2; RIP1-VEGFB (N = 6, n =  32) (D) mice. Results are displayed as % of phospho-Histone-3 (A), cleaved caspase-3 (B) or DAPI (D) stained area in relation to the tumor area. N =  number of analyzed mice, n =  number of tumors
(0.68 MB TIF)

Figure S5 Analysis of the phenotypic consequence of VEGF-B expression in RIP1-Tag2 tumors. A) Evaluation of mFatp1-3, mBik and mBmf mRNA expression by quantitative PCR in total tumors of RIP1-Tag2 (n = 5) and RIP1-Tag2; RIP1-VEGFB (n = 5) mice. The mRNA expression profiles of the indicated genes are normalized to the expression of the internal control gene ribosomal protein 19 (mRPL19). B) Oil red lipid stain of frozen pancreatic tumor sections of RIP1-Tag2 (left) and RIP1-Tag2; RIP1-VEGFB (right). The inset shows oil red stain of a liver section. Scale bar: 100 µm.
(4.07 MB TIF)

Figure S6 Characterization of the phenotype of tumors derived from Vegfb-deficient RIP1-Tag2 tumors. A) Quantification of the number of angiogenic islets in 12-weeks old RIP1-Tag2; Vegfb+/- (n = 14) and RIP1-Tag2; Vegfb-/- (n = 8) mice. B) Quantification of tumor cell proliferation in lesions from RIP1-Tag2; Vegfb+/- (n = 27) and RIP1-Tag2; Vegfb-/- (n = 26) mice. C) Quantification of the number of infiltrating immune cells in lesions (n = 24 for each genotype) from RIP1-Tag2; Vegfb+/- and RIP1-Tag2; Vegfb-/- mice depicted as average +/- standard deviation. Results are displayed as % of stained area to tumor area.
(0.33 MB TIF)

Materials and Methods S1 (0.03 MB DOC)
