# Supplementary figures and images for: Suppressive Effects of Vascular Endothelial Growth Factor-B on Tumor Growth in a Mouse Model of Pancreatic Neuroendocrine Tumorigenesis
Source: PLoS One. 2010 Nov 24;5(11):e14109. doi: 10.1371/journal.pone.0014109 (PMC2991338; doi:10.1371/journal.pone.0014109)

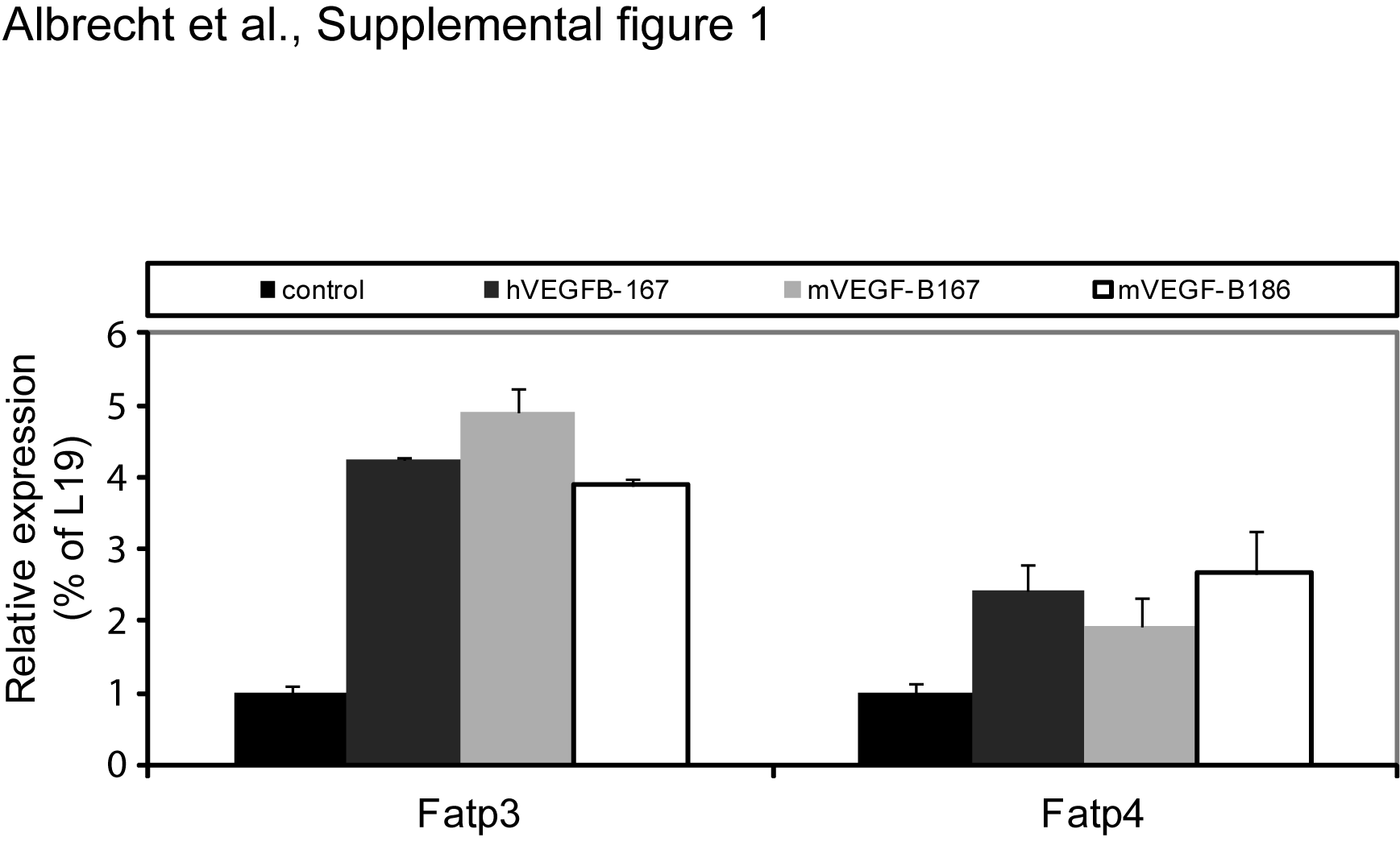

Supplement: Figure S1 — Comparison of the ability of mouse and human VEGF-B to activate VEGFR-1 downstream target gene transcription. Quantitative RT-PCR determination of the induction of FATP3 and FATP4 mRNA by mouse pancreatic islet endothelial cells (MS1) following 24h of stimulation by control, human VEGF-B167, or mouse VEGF-B167 and VEGF-B186. (0.13 MB TIF) [file pone.0014109.s001.tif]

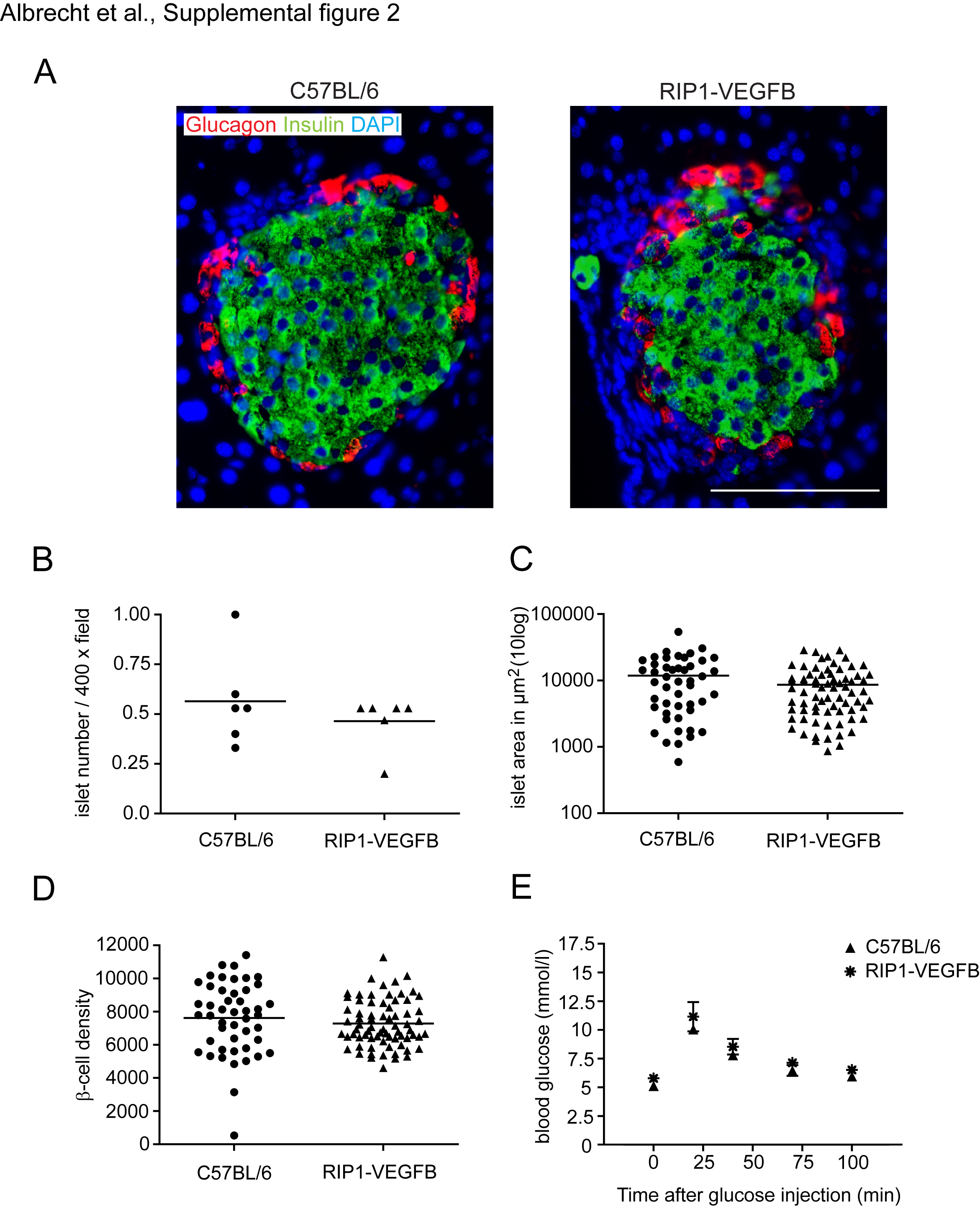

Supplement: Figure S2 — Characterization of the pancreatic islet architecture in RIP1-VEGFB mice. A) Pancreatic sections of control C57BL/6 (left) and of RIP1-VEGFB mice (right) stained for glucagon and insulin to examine islet architecture. Nuclei were counterstained with DAPI. Scale bar: 100 µm. B, C) Quantification of islet number (B, left), area (B, right) and Beta-cell density (C) was performed on H&E stained paraffin sections of C57BL/6 (N = 8) and RIP1-VEGFB (N = 6) mice. Determination of islet area and of Beta-cell number per islet area was done using computer-assisted image analysis. Beta-cell density is shown as nuclei per islet area in mm2. *, p = 0.0108 (Student's t-test). D) Intra-peritoneal glucose tolerance test: After 16 hours of starvation C57BL/6 (N = 6) and RIP1-VEGFB (N = 6) mice were i.p. injected with 1g glucose/kg body weight, and subsequently blood glucose levels were determined at the indicated time points. (3.68 MB TIF) [file pone.0014109.s002.tif]

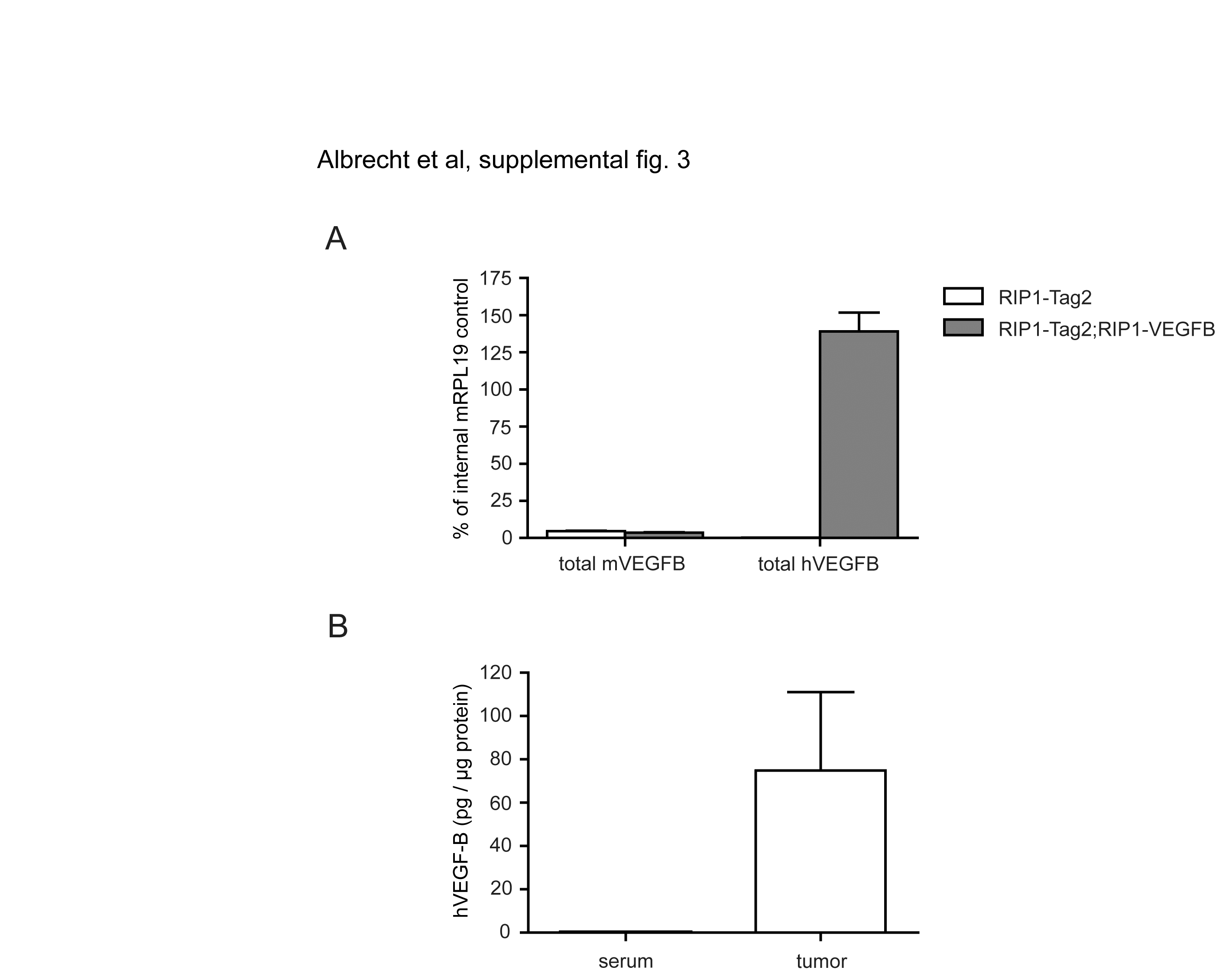

Supplement: Figure S3 — Analysis of the expression of VEGFB in RIP1-VEGFB mice. A) Quantitative RT-PCR determination of expression of mouse and human VEGF-B in tumors from RIP1-Tag2 and Rip1-Tag2; RIP1-VEGFB mice. B) Analysis of the abundance of human VEGF-B protein in serum and tumor tissue from RIP1-Tag2; RIP1-VEGFB mice using ELISA. (0.23 MB TIF) [file pone.0014109.s003.tif]

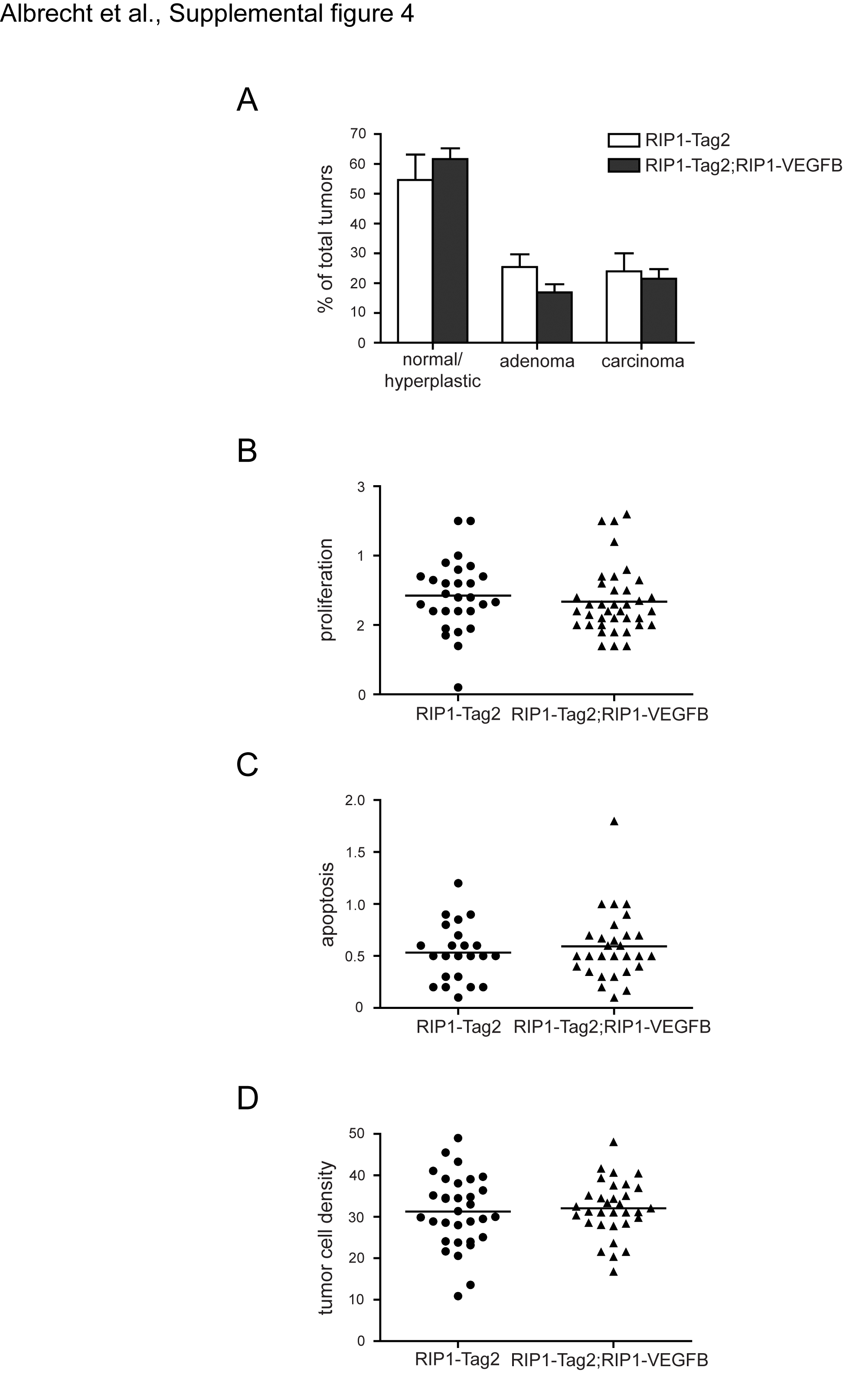

Supplement: Figure S4 — Characterization of the phenotype of tumors derived from RIP1-Tag2; RIP1-VEGFB mice. A) Staging of tumors into normal/hyperplastic islets, adenoma or carcinoma in RIP1-Tag2 (N = 6) and RIP1-Tag2; RIP1-VEGFB (N = 6) mice. B, C, D) Quantification of tumor cell proliferation in RIP1-Tag2 (N = 5, n = 28) and RIP1-Tag2; RIP1-VEGFB (N = 5, n = 38) (B) mice, of tumor cell apoptosis in RIP1-Tag2 (N = 4, n = 23) and RIP1-Tag2; RIP1-VEGFB (N = 5, n = 29) mice (C) and of tumor cell density in RIP1-Tag2 (N = 8, n = 32) and RIP1-Tag2; RIP1-VEGFB (N = 6, n = 32) (D) mice. Results are displayed as % of phospho-Histone-3 (A), cleaved caspase-3 (B) or DAPI (D) stained area in relation to the tumor area. N = number of analyzed mice, n = number of tumors (0.68 MB TIF) [file pone.0014109.s004.tif]

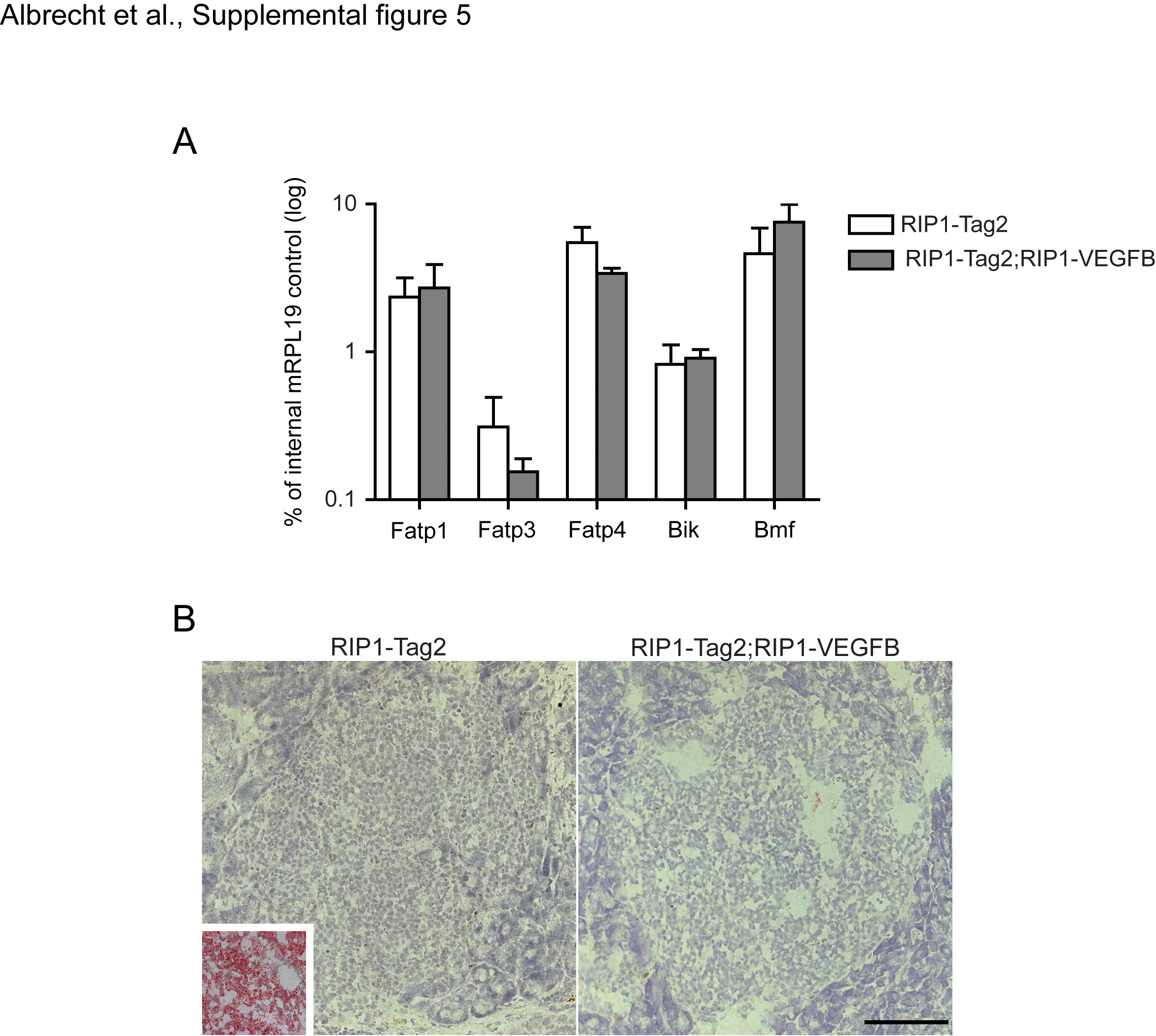

Supplement: Figure S5 — Analysis of the phenotypic consequence of VEGF-B expression in RIP1-Tag2 tumors. A) Evaluation of mFatp1-3, mBik and mBmf mRNA expression by quantitative PCR in total tumors of RIP1-Tag2 (n = 5) and RIP1-Tag2; RIP1-VEGFB (n = 5) mice. The mRNA expression profiles of the indicated genes are normalized to the expression of the internal control gene ribosomal protein 19 (mRPL19). B) Oil red lipid stain of frozen pancreatic tumor sections of RIP1-Tag2 (left) and RIP1-Tag2; RIP1-VEGFB (right). The inset shows oil red stain of a liver section. Scale bar: 100 µm. (4.07 MB TIF) [file pone.0014109.s005.tif]

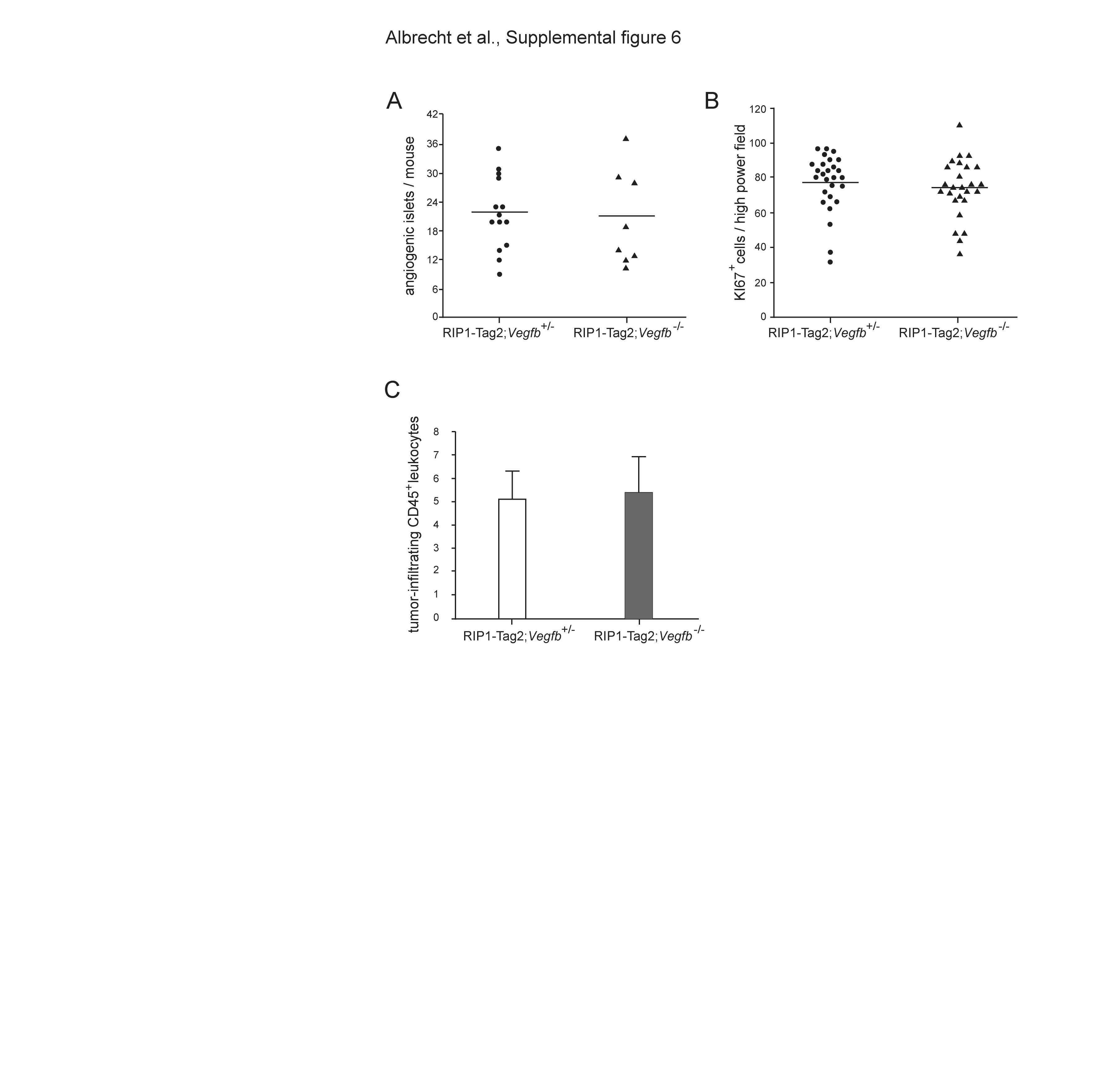

Supplement: Figure S6 — Characterization of the phenotype of tumors derived from Vegfb-deficient RIP1-Tag2 tumors. A) Quantification of the number of angiogenic islets in 12-weeks old RIP1-Tag2; Vegfb+/- (n = 14) and RIP1-Tag2; Vegfb-/- (n = 8) mice. B) Quantification of tumor cell proliferation in lesions from RIP1-Tag2; Vegfb+/- (n = 27) and RIP1-Tag2; Vegfb-/- (n = 26) mice. C) Quantification of the number of infiltrating immune cells in lesions (n = 24 for each genotype) from RIP1-Tag2; Vegfb+/- and RIP1-Tag2; Vegfb-/- mice depicted as average +/- standard deviation. Results are displayed as % of stained area to tumor area. (0.33 MB TIF) [file pone.0014109.s006.tif]
