# Supplementary material for: Suppressive Effects of Vascular Endothelial Growth Factor-B on Tumor Growth in a Mouse Model of Pancreatic Neuroendocrine Tumorigenesis
Source: PLoS One. 2010 Nov 24;5(11):e14109. doi: 10.1371/journal.pone.0014109 (PMC2991338; doi:10.1371/journal.pone.0014109)
Supplement: Materials and Methods S1 — (0.03 MB DOC) [file pone.0014109.s007.doc]

**Supplemental methods**

***Endothelial cell stimulation***

Mile Sven-1 cells (ATCC) were grown according to the provided instructions. MS-1 cells were treated with 100 ng/mL of mVEGF-B_186_, mVEGF-B_167_, hVEGF-B_167_ (R&D systems, Minneapolis, MN) as previously described (see reference 13).

***Histopathological analysis***

The number of islets was counted on hematoxylin/eosin (H&E)-stained pancreatic sections of C57BL/6 and RIP1-VEGFB mice and related to the total pancreas area. The average islet area was measured using Image J software (Rasband, W.S., ImageJ, U. S. National Institutes of Health, Bethesda, Maryland, USA, http://rsb.info.nih.gov/ij/, 1997-2007) and β-cell density was calculated by determination of the nuclei number per islets in relation to the islet area.

To examine the islet architecture, paraffin-embedded pancreatic sections of C57BL/6 and RIP1-VEGFB mice were stained for insulin (DAKO) and for glucagon (Santa Cruz).

Tumor grading was performed on H&E stained pancreatic section of RIP1-Tag2 and RIP1-Tag2; RIP1-VEGFB mice. Thereby, all tumors found were staged according their appearances into hyperplastic islets, adenoma and carcinoma in a blindfold manner.

Tumor cell proliferation and apoptosis was determined by staining frozen pancreatic sections for cleaved caspase-3 (Cell Signaling Technology) and for phospho-Histone H3 (Millipore-Upstate) respectively. Subsequent quantification was performed using Image J software and the results are displayed as % of stained intratumoral area related to tumor area. Lipids were visualized with Oil-red-O staining.

***Intra-peritoneal glucose tolerance test***

C57BL/6 and RIP1-VEGFB mice between 7 and 23 weeks of age were starved for 16 hours prior to intraperitoneal injection of 1g D-(+) glucose anhydrous (Sigma)/kg body weight. Subsequently, 0, 25, 50, 75 and 100 min after injection blood glucose levels were measured by tail vein blood sampling using the Accu-Check Comfort device and test stripes (Roche Diagnostics Corporation, Indianapolis). Data shown are the means ± standard deviation.

***Quantitative RT-PCR***

Total RNA was extracted from isolated pancreatic tumors of RIP1-Tag2 and RIP1-Tag2; RIP1-VEGFB mice using TRIzol reagent. After DNase treatment of the RNA, first-strand cDNA was synthesized with M-MLV reverse transcriptase RNAse-H (Promega). Quantitative PCR for mouse Fatp1, 3, 4, Bik and Bmf transcripts was done on ABI Prism 7000 (Applied Biosystems) using the SYBR-green PCR MasterMix (Applied Biosystems) and normalized versus the mouse ribosomal protein 19 (mRPL19) transcript. The following primers were used:

mRPL19: ATCCGCAAGCCTGTGACTGT and TCGGGCCAGGGTGTTTTT

mFatp1: CGGCGTTCTGTGTGTACG and CCGAACACGAATCAGAACAG

mFatp3: CCTCGGTTTCTCAGGCTCCA and CTGTACCGGGCAGGTGTGA

mFatp4: CTATGACTGCCTCCCCCTCT and AGTCATGCCGTGGAGTAAGC

mBik: ACGTGGACCTCATGGAGTG and GCAGACACAGGTCCATCTCA

mBmf: TGTCATGCTGCCTTGTGG and AGGGAGAGGAAGCCTGTAGC

mVEGF-B: CCCAGCCACCAGAAGAAA and ACATTGCCCATGAGTTCCAT

hVEGF_B: TGTCTCCCAGCCTGATGC and GGCAGGTAG CGCGAGTAT

***ELISA***

Quantification of hVEGF-B in the sera and in the tumors of RIP1-Tag2, RIP1-VEGFB mice was performed with the ELISA Kit for hVEGFB (Hölzel Diagnostika) according to the manufacturer`s instructions.

The sera and tumor lysates were prepared as follows. The blood samples were incubated at RT to allow clotting before centrifugation for 20 min at 1000xg. The serum was removed and used freshly or stored at -80°C for later use. Tumors were isolated from the pancreas and homogenized in ice-cold PBS containing protease inhibitors with a glass homogenizer. The resulting suspension was freeze-thawn twice and subsequently cleared by centrifugation for 5 min at 5000xg. The supernatant was removed and assayed immediately or stored at -80°C.
